# Investigation and characterization of human gut phageome in advanced liver cirrhosis of defined etiologies

**DOI:** 10.1186/s13099-022-00482-4

**Published:** 2022-02-15

**Authors:** Mohadeseh Naseri, Fahimeh Palizban, Abbas Yadegar, Mohsen Khodarahmi, Hamid Asadzadeh Aghdaei, Hamidreza Houri, Javad Zahiri

**Affiliations:** 1 Bioinformatics and Computational Omics Lab (BioCOOL), Department of Biophysics, Faculty of Biological Sciences, Tarbiat Modares University, Tehran, Iran; 2 Laboratory of Complex Biological Systems and Bioinformatics (CBB), Institute of Biochemistry and Biophysics (IBB), University of Tehran, Tehran, Iran; 3 Foodborne and Waterborne Diseases Research Center, Research Institute for Gastroenterology and Liver Diseases, Shahid Beheshti University of Medical Sciences, Shahid Arabi Ave., Yemen St., Velenjak, Tehran, Iran; 4 Medical Imaging Center, Karaj, Alborz Iran; 5 Basic and Molecular Epidemiology of Gastrointestinal Disorders Research Center, Research Institute for Gastroenterology and Liver Diseases, Shahid Beheshti University of Medical Sciences, Tehran, Iran; 6 Department of Neuroscience, University of California, San Diego, 9500 Gilman Drive, La Jolla, CA 92093-0662 USA

**Keywords:** Liver cirrhosis, Phageome, Metagenomics, Nonalcoholic fatty liver disease

## Abstract

**Background:**

Liver cirrhosis is a major public health problem, accounting for high rates of morbidity and mortality worldwide. The cirrhosis etiology is a broad and essential step in planning a treatment strategy. Many recent studies have documented that gut microbiome alterations play a vital role in the development and progression of cirrhosis and its complications. Nevertheless, there is insufficient data on the correlation between liver cirrhosis and gut phageome alterations in patients with advanced liver diseases. This study aimed to analyze the taxonomic structure and functional attributes of the gut phageome in six different etiologies of advanced liver cirrhosis.

**Methods:**

We first retrieved metagenomic sequencing data from three datasets of fecal samples taken from cirrhotic patients with various etiologies. Subsequently, several bioinformatics pipelines were used to analyze bacteriophage composition and determine their functionality.

**Results:**

A gene catalog of 479,425 non-redundant genes was developed as a reference to measure gene prevalence. The results of the analysis revealed a few significant differences among the cohorts at the phage species level. However, the alternations were more evident as there were more in-depth analyses of the functional resolution of the gut phageome.

**Conclusions:**

Our findings suggest that the functional analysis of the gut phageome would provide meaningful markers to predict the progression of liver cirrhosis and facilitate the development of novel treatment approaches.

**Supplementary Information:**

The online version contains supplementary material available at 10.1186/s13099-022-00482-4.

## Introduction

The human gut microbiome comprises an extremely diverse and complex community of microorganisms and is a highly dynamic ecosystem affecting the host health status and accelerating the disease progression [1]. There has been an increased interest in understanding the role of the gut microbiome in developing metabolic disorders, with some studies sparing efforts to elucidate the functional significance of the microbiome in the progression of liver diseases [2, 3]. Nowadays, the metagenomics analysis of the human-associated microbiome provides a rich set of microbial features for prediction and biomarker discovery in the context of human diseases and health conditions [4]. Among these diseases, liver disorders annually account for about 2 million deaths ⁠worldwide, in which more than 1 million cases are diagnosed with liver cirrhosis complications [5]. Liver cirrhosis is an irreversible end result of a long-lasting clinical course of several chronic liver diseases, which are commonly a consequence of long-term alcohol abuse, infection with hepatitis viruses, nonalcoholic fatty liver disease, etc. [6, 7]. More importantly, the human gut microbiome seems to play a vital role in the development and progression of cirrhosis [8, 9]. For example, Loomba et al. conducted a study to identify the gut microbiome signatures of advanced fibrosis in patients with Nonalcoholic Fatty Liver Disease (NAFLD) [10]. Similarly, Zhao et al. characterized the gut microbiota composition and the functional annotations of Chinese children and adolescents with NAFLD [11].

To date, most microbiome studies have focused on the bacterial composition of the human gut, and less attention has been paid to the detailed analysis of the whole community of viruses and bacteriophages, henceforth called virome and phageome, respectively [12, 13]. More recently, the contribution of phage populations to the gut microbiome ecology and their effects on human health and diseases has been to be deciphered. Emerging data indicate that gut-associated phages can play a significant role in gastrointestinal physiology by regulating bacterial density, maintaining biodiversity, and controlling network interactions among the gut bacterial communities [14–16].

Previously, healthy individuals were assumed to have a stable gut phageome, which predominantly constituted of non-enveloped double-stranded DNA (dsDNA) *Caudovirales* or single-stranded DNA (ssDNA) *Microviridae*, as well as ssDNA filamentous phages, i.e., *Inoviridae*, reproduced by chronic infection without killing their host [17–19]. Interestingly, any compositional changes in the gut phageome could be associated with a variety of gut-related and systemic disorders such as inflammatory bowel diseases (IBD) [14], AIDS [20], and even malnutrition [21]. For example, an in-depth metagenomics analysis revealed a significant increase in the number of enteric *Caudovirales* phages in patients with Crohn’s disease (CD) and ulcerative colitis (UC) [14]. Ma et al. carried out the first study exploring the large and diverse communities of gut phages in type II diabetes and declared the significance of the phageome in type II diabetes [22]. However, these investigations address a key obstacle: there is currently no single recognized database of annotated virus genome sequences, and de novo prediction of virus sequences from metagenomic assemblies has remained a remarkable challenge.

It is worth mentioning that little attention has been paid to detecting the role of gut phageome in liver diseases; hence, future studies need to delve into the characteristics of the gut phageome in various liver disorders. Moreover, access to a reference gene catalog is inevitable to deeply investigate microbial environments such as the human gut. Accordingly, there have been several attempts to construct specific human microbiome gene catalogs containing several types of microorganisms, mainly bacterial species [23]. In this regard, a better understanding of the phage content of the human gut depends on access to a comprehensive gene catalog of the gut phageome. In the present study, we adopted three bioinformatic strategies to identify the large scaffolds of phage origin to investigate the structural and functional composition of gut phageome in several etiologies of liver diseases. This is the first study to correlate the gut phageome with liver cirrhosis to provide valuable insights into the design of novel phage-related markers in monitoring liver disease progression and its treatment.

## Methods

### Input data characteristics and collection procedure

In this study, three previously published metagenomic datasets were employed for the analyses. In this regard, three metagenomic datasets were obtained from the National Center for Biotechnology Information’s (NCBI’s) Sequence Read Archive (SRA) to investigate the gut phageome of cirrhotic patients from structural and functional aspects deeply. The total number of participants with liver cirrhosis in these three studies was 139, of whom 98 samples belonged to the Chinese population (PRJEB6337). The dataset of the Chinese population was categorized as alcohol-induced cirrhosis (Chinese ALC, n = 10), hepatitis B virus-related cirrhosis (Chinese HBV, n = 30), a combination of hepatitis B virus and alcohol-related cirrhosis (Chinese ALC-HBV) (n = 24), and other etiologies (e.g., hepatitis C, E, and D virus, etc.; n = 34) [24]. The metagenomic dataset, which was reported by Tyakht et al. and obtained from 27 Russian patients with alcohol-related cirrhosis, was included as the Russian ALC cohort (PRJNA373901) [25]. Finally, 14 nonalcoholic fatty liver disease (NAFLD) patients with advanced-stage fibrosis, who were evaluated by Loomba et al., were included as the NAFLD cohort (PRJEB18041) [10]. The quality of the sequencing reads was verified using FastQC software before further analysis [26]. Furthermore, the host residual genome content was checked in gut metagenomic data by aligning the reads to human reference genome hg38 using Bowtie2 [27]. Finally, due to the batch effects of different groups, the remove batch effect function was considered from the limma package [28].

### Phage catalog construction

**Fig. 1:**
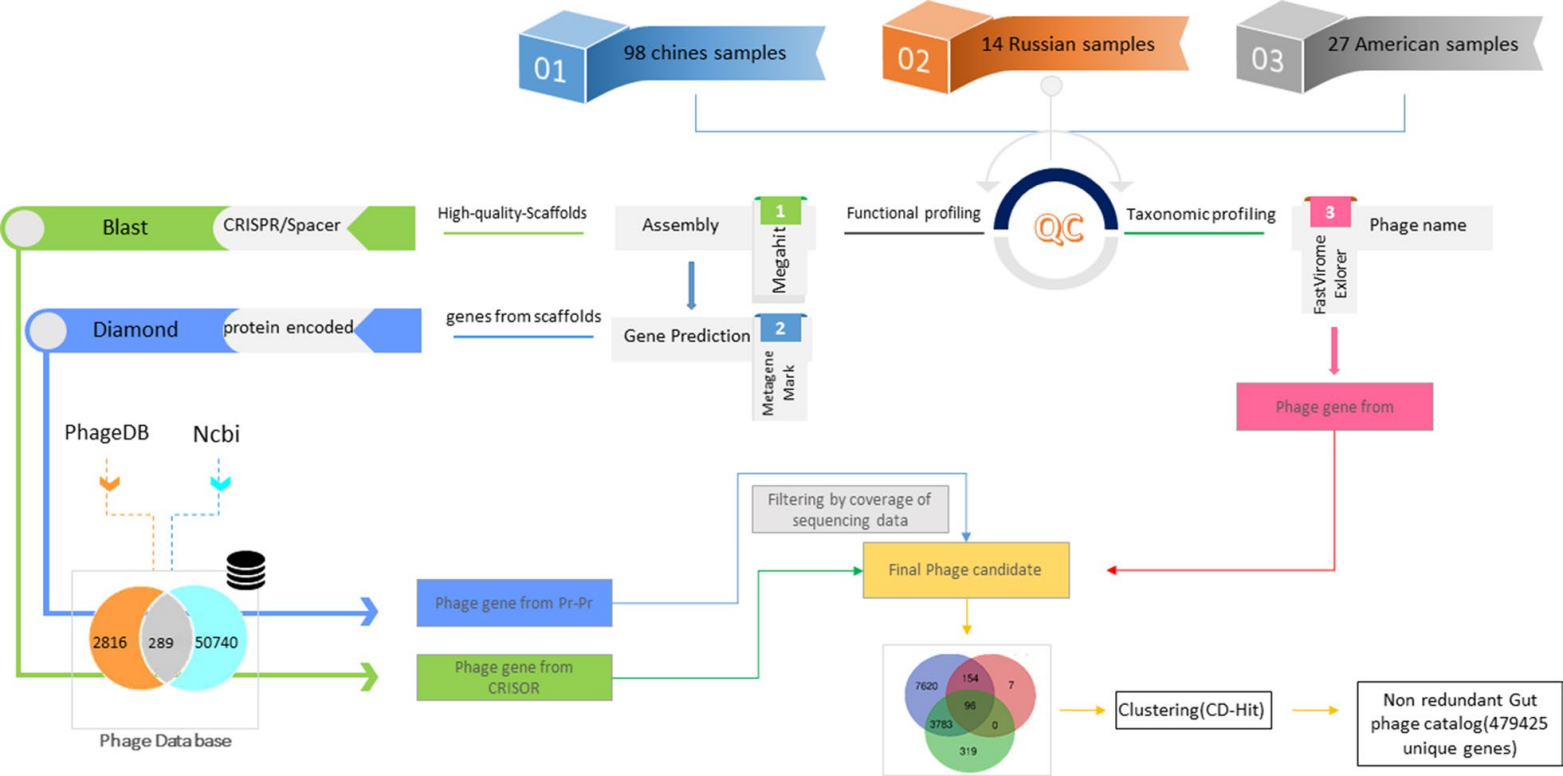
A schematic overview of the method proposed for developing phage gene-catalog. The pipeline used three strategies to construct the gut phage catalog to facilitate the taxonomic and functional analysis of the gut phageome in liver cirrhosis

The main objective of this study was to construct the gut phage catalog facilitating the structural and functional analysis for the phageome of liver cirrhosis. To this end, the proposed approach encompassed three different strategies, as illustrated in Fig. 1. Furthermore, the high-quality metagenomic reads were analyzed by the FastViromeExplorer tool [29] to determine the gut phage composition in various etiologies of cirrhosis. The genetic sequences of known strains were identified by FastViromeExplorer and retrieved from the NCBI Refseq. Furthermore, we spared our efforts to determine all spacers from the 139 assembled metagenomic samples to improve the generality of our catalog. In this regard, metagenomic assembly was performed by MEGAHIT [30], and CRISPRFinder [31] was then used to predict all potential spacers in a unified assembled file. These spacers were mapped to a locally developed database of all available phages, which was made by integrating the phage sequences of NCBI and phage database (https://phagesdb.org/). We also prepared a comprehensive database encompassing the genome of all known phages from distinct resources. Accordingly, 50,740 and 2816 phage genomes were obtained from NCBI and phagesDB, respectively. This database would provide the grounds for the more accurate and efficient investigation and identification of gut phageome. Further, MetaGeneMark with default flags was used to generate the nucleic acid and amino acid sequences related to the predicted genes from the assembled file. Moreover, the protein sequences of the predicted genes were mapped to the previously mentioned local phage database by the DIAMOND alignment tool [32]. Finally, all genes identified by the three strategies were integrated into one single gene catalog, and CD-HIT⁠ [33] was then applied to remove redundant genes (with a 90% similarity threshold).

### Phage community analysis and taxonomic profiling

Before any phage community analysis, several filtrations were applied on phage species, genus, and family resolutions to eliminate the wrong and pseudo correlations. Accordingly, a taxon with < 0.001 prevalence in more than five samples was removed. The Vegans [34] and Phyloseq R [35] packages were used to measure the dissimilarity and alpha diversity (i.e., ACE, Shannon, and Simpson indices), respectively. The differences among the mentioned variables in several phenotypes were detected by the ANOVA test. Further statistical analysis and visualization steps were conducted using ggplot2 R packages [36].

### Functional analysis of human gut phageome

We developed a phage gene catalog to identify the putative biochemical interaction networks between the gut phageome and microbiome. Accordingly, the raw metagenomic samples were mapped to the gene catalog by the MOSAIK aligner [37], and samtools idxstats [38] were then used to determine the frequency of each covered gene in each sample. Differentially prevalent genes in each cohort were normalized and extracted by the edgeR package [26]. In the next step, we investigated the gene sets specific for each etiology based on their abundance and statistical test analysis. Accordingly, we found the specific and significantly related gene sets for each etiology by using edgeR based on the fold change and adjusted *p*-value. Then common genes among different groups were omitted and the rest of them were extracted and assigned as an etiology-specific gene set. Afterward, the GhostKOALA tool [39] was applied to detect the KEGG orthology of the genes.

### Gene annotation of the differentially prevalent genes

In addition to the KO analysis, the functional annotation of the genes extracted by edgeR was performed by comparing the genes in different databases, including the GenBank, COG [40], eggNOG 4.5 [41], and Pfam [42] using RPS-BLAST [43] with an e-value cutoff of 1e−10. The annotation results were visualized by FuncTree2 [44].

## Results

### Phage gene catalog construction and core phageome in liver cirrhosis

**Fig. 2:**
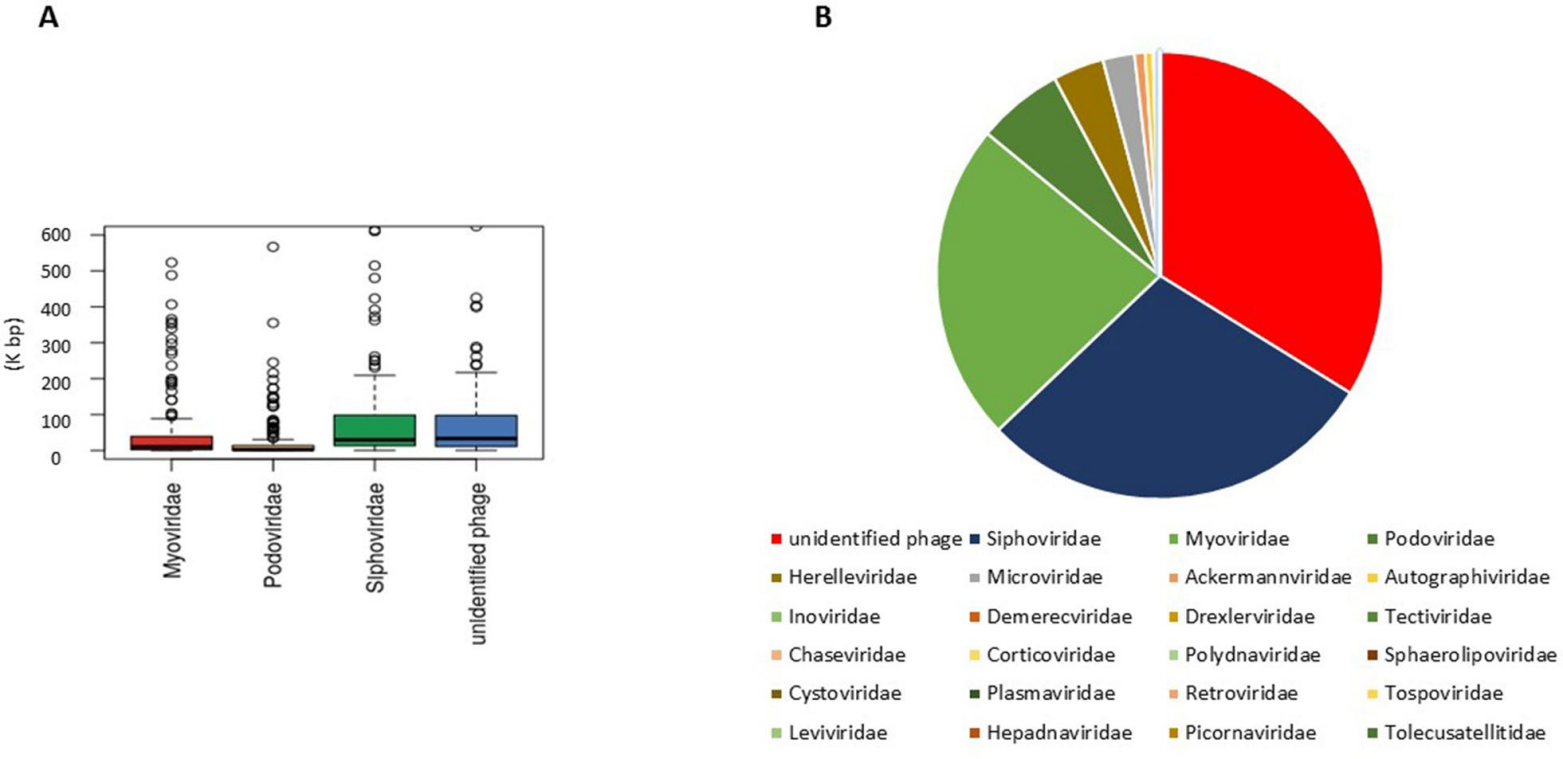
**A** Length range of the genomes mapped in different phage families, **B** The most prevalent phage families in constructed phage gene-catalog

Before applying the structural and functional analysis, a phage gene catalog of distinct etiologies of liver cirrhosis was developed in this study. Using three bioinformatic strategies, we recognized large phage scaffolds from the assembled scaffolds of the metagenomic data, including 479,425 non-redundant genes (the phage scaffolds were searched by (1) spacers of CRISPRs, (2) the known scaffolds were identified by FastViromeExplorer, and (3) the scaffold encoded proteins homologous to the proteins from a locally database of phages). As shown in Fig. 2A, the highest proportion of genes (35%) in the catalog belonged to unclassified phages, followed by the phage genes of the family *Siphoviridae* (30%). The unclassified phage scaffolds of the catalog could be an appropriate source for further analysis to detect and propose novel phage genes and strains. The large phage scaffolds ranged from 18 to 257 kb in length, consistent with the long-range of phage genomes in the family *Siphoviridae* (Fig. 2B).

**Fig. 3:**
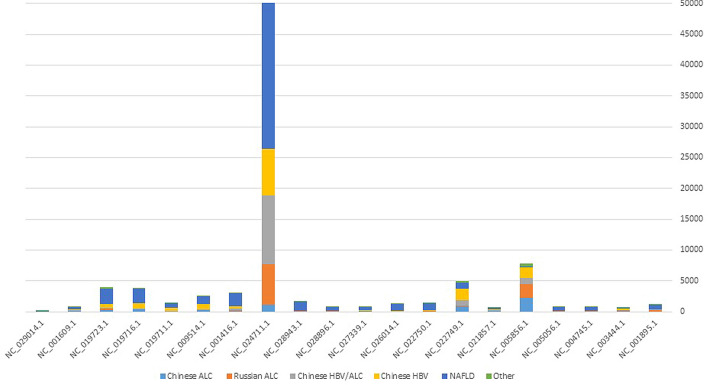
Frequency distribution of each phage belonging to core phageome among different etiologies of liver disorders. Uncultured crAssphage (NC_024711.1) was the most prevalent phage in the core phageome of cirrhotic patients

This study investigated the core phageome of distinct etiologies of liver cirrhosis using three bioinformatic strategies. Although most of the detected large phage scaffolds were unique to each sample, 21 phage species were found to be common among all cohorts and defined as the core phageome in cirrhosis, as existed in more than two-thirds of the fecal samples of cirrhotic patients (Fig. 3). Among the 21 phages, uncultured crAssphage (NC_024711.1) was the most prevalent strain in all cohorts.

### Enteric phage diversity and gut phageome composition in various cirrhotic groups

**Fig. 4:**
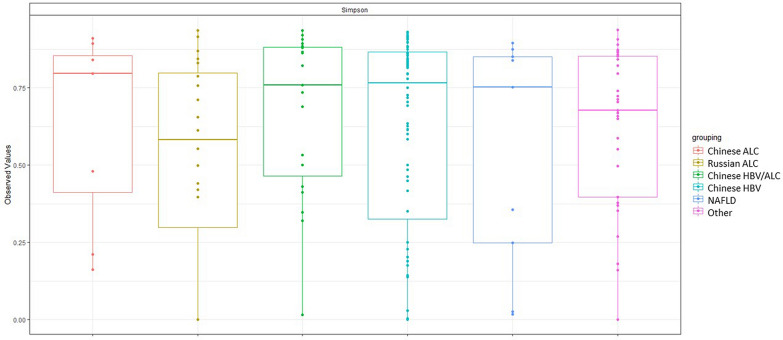
Bacteriophage population α-diversity (Simpson index) in cirrhotic patients with various etiologies

To find any specific alternation in the gut phageome among cirrhotic patients with an individual etiology, we first tried to identify the phage diversity in the cirrhotic cohorts with various etiologies. According to the FastViromeExplorer, 258 phage strains were unique to only one etiology. Increasing or decreasing α-diversity in phage composition among the different cirrhotic groups was examined using the Simpson index. Phage richness was not statistically different among the cirrhotic cohorts; however, there was a slight nonsignificant tendency for reduction of α-diversity in the Chinese ALC cohort by reduced Simpson index (Fig. 4).

**Fig. 5:**
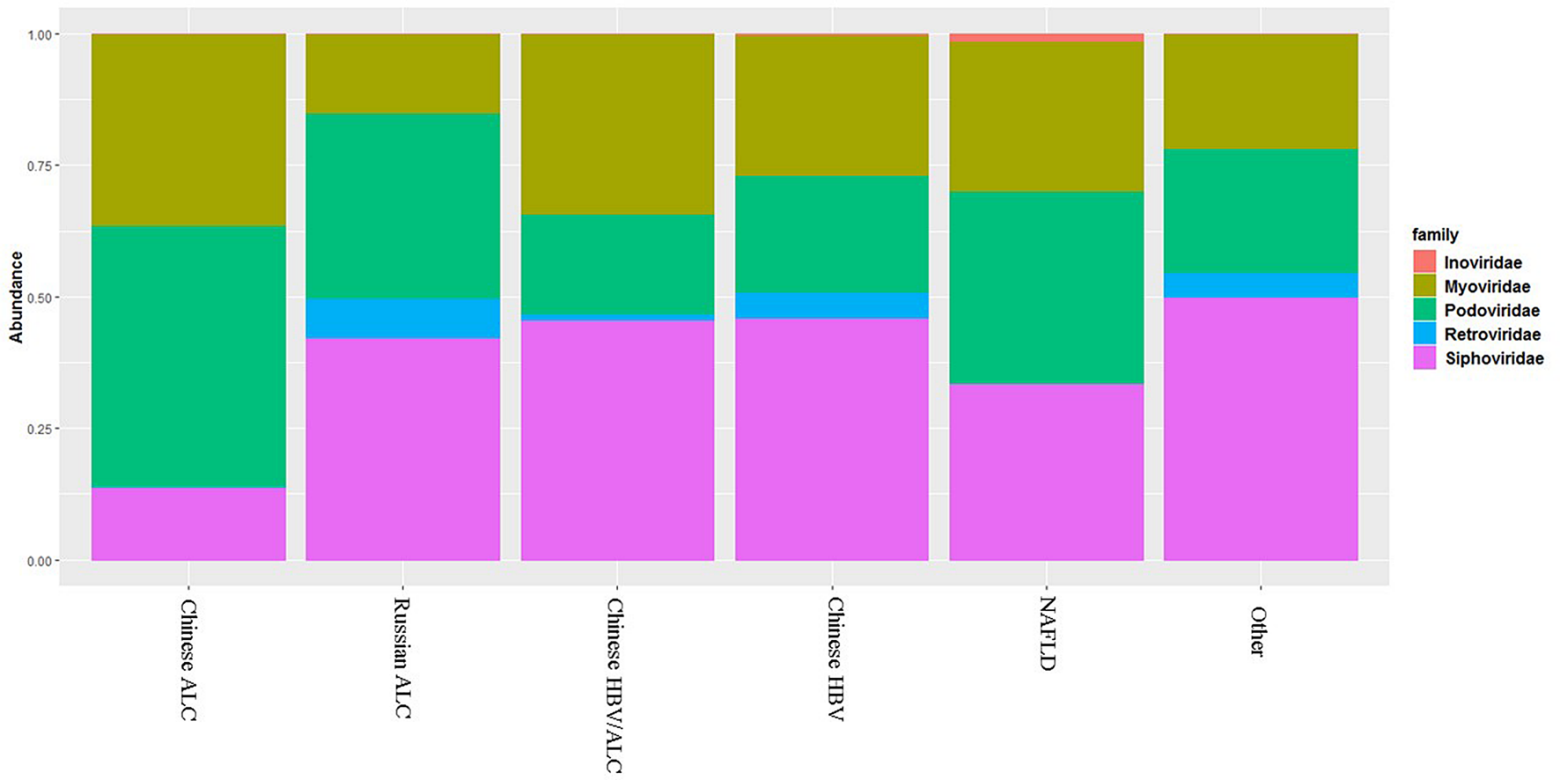
Comparing the relative frequency of dominant bacteriophage families in different cirrhosis cohorts

The relative frequency analysis at the family level (with 14 different families) revealed *Siphoviridae* as the most prevalent family in all cirrhotic cohorts, followed by *Podoviridae* and *Myoviridae* (Fig. 5). Individually, the analyses indicated that the *Retroviridae* family had been eliminated in the Chinese ALC and NAFLD groups. Moreover, post hoc pairwise analysis indicated that *Introviridae* showed a higher relative prevalence in the NAFLD group compared to the other groups. An analysis of the relative prevalence of phage taxa among Chinese cohorts revealed no significant difference among them at the family level regarding the etiology of cirrhosis, implying that the gut phageome could be strongly affected by geographical, racial, social, and dietary factors.

**Fig. 6:**
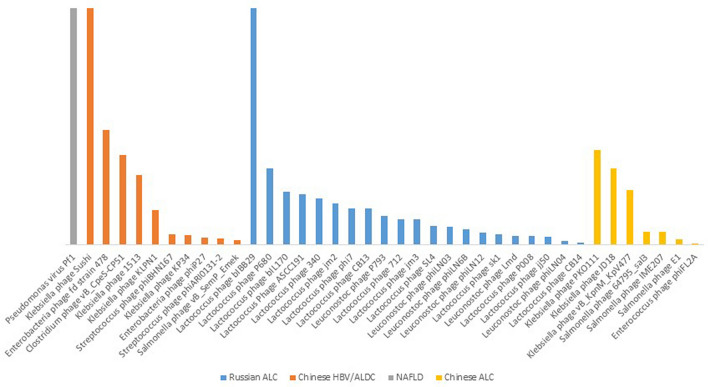
Etiological-specific enteric bacteriophages among cirrhotic patients. Each bar plot indicates the frequency of a bacteriophage species detected in an individual etiology exhaustively

In the next step, we assessed phage species detectable only in one cirrhotic cohort to identify the presence or the absence of individual phage species and find a phageome signature in each etiology. Our analysis revealed that the Pseudomonas virus Pf1 phage, which belonged to the *Inoviridae* family, was only detected in the NAFLD group. Remarkably, *Leuconostoc* and *Lactococcus* phages belonging to the family *Siphoviridae* were identified as the individualized phage strains in the Russian ALC group. Interestingly, the results of the present study also indicated that most of the phages in the Chines cohorts were specific to the *Enterobacteriaceae* hosts, particularly *Klebsiella* spp. Figure 6 illustrates the individual phage richness in each cirrhotic group exhaustively.

**Fig. 7:**
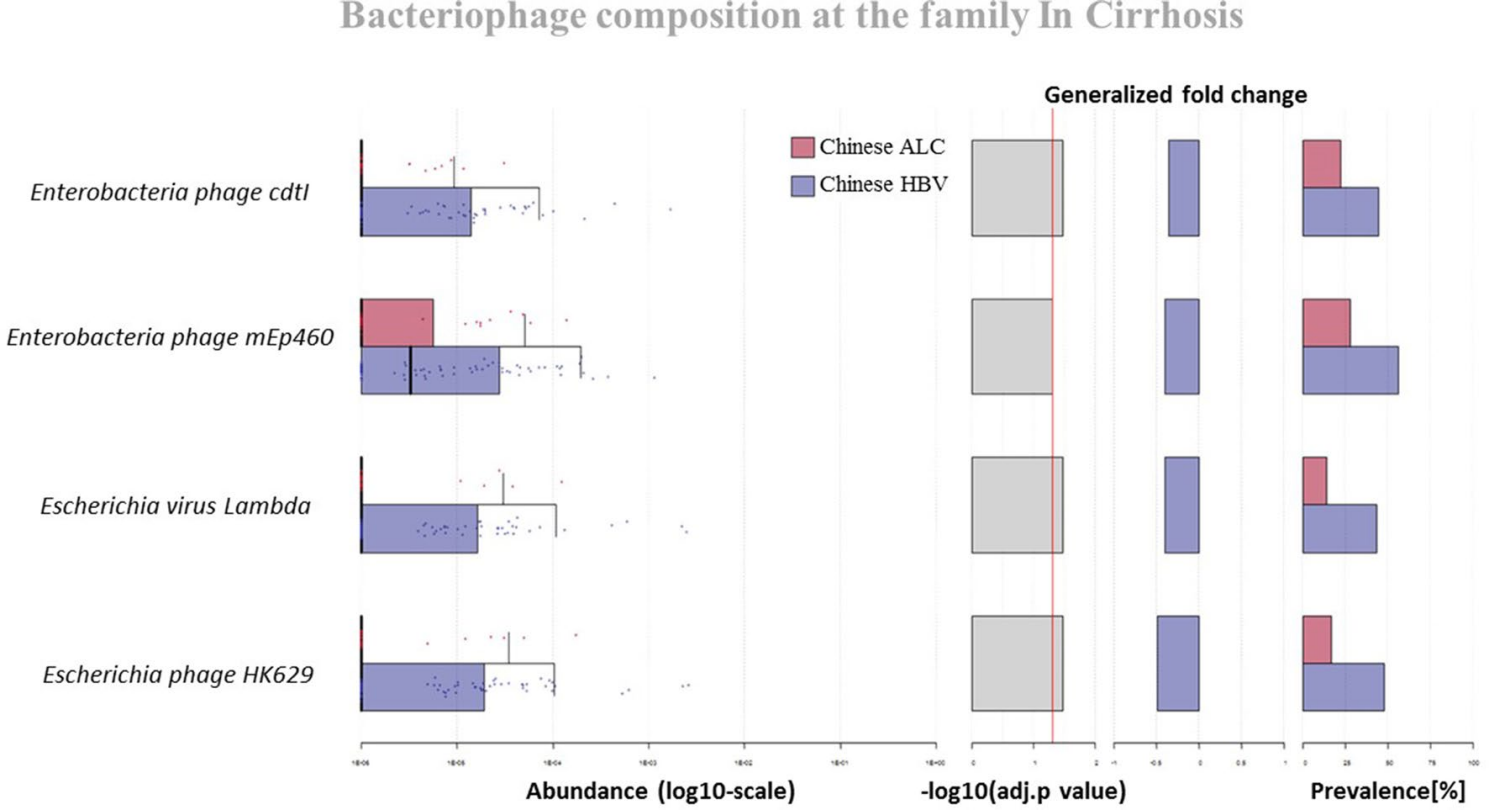
Bacteriophages with different frequencies in two groups of Chinese cirrhotic patients (namely Chinese ALC and Chinese HBV cohorts), according to genus resolution

To investigate phage compositional differences between Chines cirrhotic patients with different etiologies (i.e., Chines ALC and Chines HBV), we analyzed the prevalence of phage species unique to each group. According to the findings, four phages of the family Siphoviridae with completely different prevalence rates were identified in the Chines HBV and Chines ALC cohorts. These phages belonged to the unclassified Siphoviridae group (Enterobacteria phage cdtI and Enterobacteria phage mEp460) and the genus Lambdavirus (Escherichia virus Lambda and Escherichia phage HK629). As presented in Fig. 7, all four phages had higher frequencies in the Chinese HBV group. Moreover, we compared the NAFLD and ALC (i.e., Chinese ALC and Russian ALC) groups regarding the composition of the phage community at the species level. Notably, the uncultured phage crAssphage from the *Podoviridae* family was significantly higher in the NAFLD group compared to the ALC cohorts.

### Functional annotation of the enriched gene sets

We also aimed to investigate functional markers as suitable predictive factors for each case of liver cirrhosis with different etiologies. Accordingly, by mapping each of the 139 samples to the constructed phage gene catalog, the prevalence of each gene in each sample was measured and then normalized based on the TMM normalization method of the edgeR package. Additional files 1, 2, 3, 4 and 5 present the frequencies of specific genes for each liver cirrhosis etiology.

**Fig. 8:**
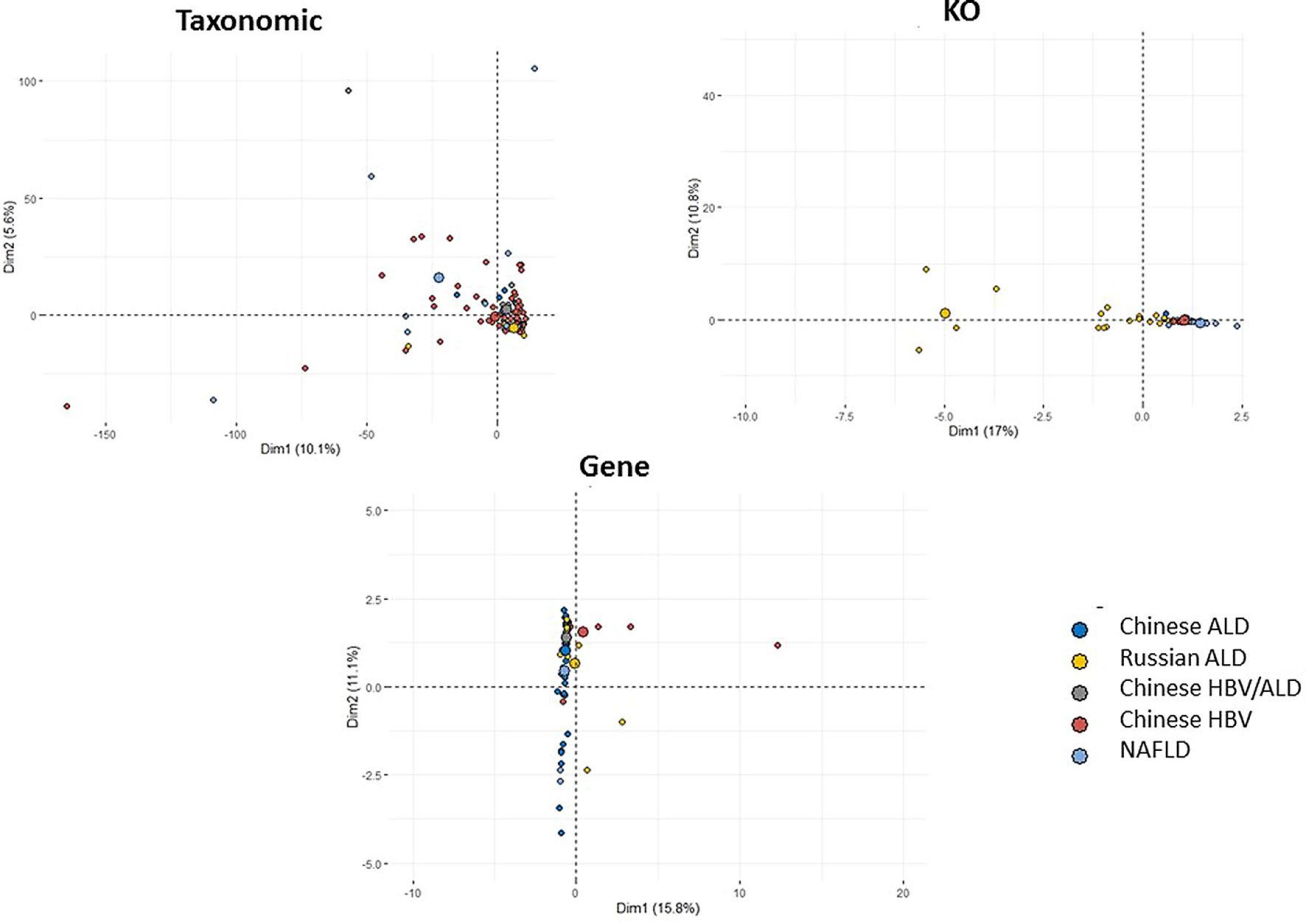
PCA analysis of three resolutions, including taxonomic, gene, and KO analysis. As it was concluded, KO is a better discriminating factor two other features are compared

**Fig. 9:**
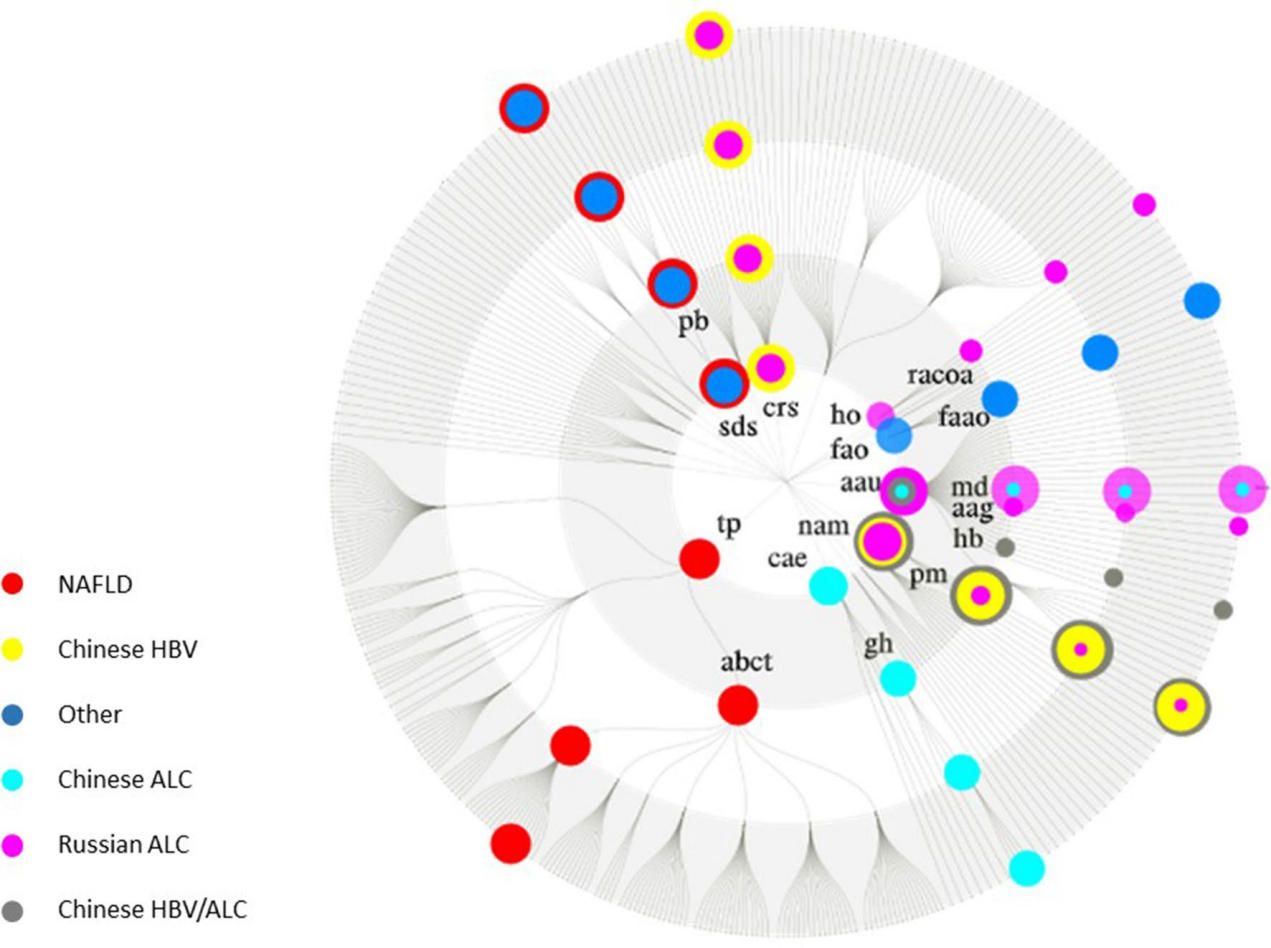
Functional annotation based on KO analysis performed by FuncTree2. The KEGG biologic categories are as follows: (1) cellular response to stress (crs); (2) Amino acid utilization biosynthesis metabolism (aau): methionine degradation (md), alanine, aspartate and glutamate metabolism (aag); (3) nucleic acid metabolism (nam): pyrimidine metabolism (pm); (4) carbohydrate active enzyme (cae): glycoside hydrolase (gh); (5) fatty acid oxidation (fao): fatty acid alpha-oxidation (faao); (6) homoacetogenesis (ho): reductive acetyl coenzyme A pathway (racoa); (7) saccharide and derivated synthesis (sds): polysaccharide biosynthesis (pb), (8) transporters (tp): ABC transporter (abct); The KO frequency was determined and plotted (size = relative frequency of KOs in each plot)

Furthermore, the KEGG orthology (KO) analysis was carried out to deeply understand the exact biological mechanisms in the phage community of the human gut in different liver cirrhosis. To this end, the specific mapped genes in each phenotype were firstly extracted, and GkostKOALA was then used to determine the KOs of each phenotype. According to our analysis, K00986 and K06905 were the most common in all cirrhotic groups, which were related to RNA-directed DNA polymerase and uncharacterized protein, respectively. Comparatively, the Russian ALC and NAFLD phenotypes had the most distinct KOs with the frequency of 87 and 36 numbers, respectively (Additional file 6). More importantly, regarding KOs, the samples belonging to the same phenotype had the highest similarity. As shown in Fig. 8, KO can be a proper discriminating feature compared to taxonomic and other genomic features in classifying different etiologies of liver cirrhosis. According to the PCA analysis, taxonomic features cannot be proper markers to classify different etiologies of liver disorders. However, the KO functional analysis of the gut phageome could be a practical approach to discriminate liver cirrhosis with various etiologies. Figure 9 shows the functional annotation of the phageome genes identified in the cirrhotic patients based on KO analysis. Carbohydrate active enzyme was specific KEGG pathway in Chinese ALC group, while homoacetogenesis was unique pathway for Russian ALC casese. Moreover, transporters and saccharide synthesis pathway were spesefic KOs in NAFLD cohort. Nucleic acid metabolism and amino acid utilization biosynthesis metabolism were the common pathways identified in all of cirrhotic groups. Additional file 7: Figure S1 provide further information that revealed the results of FuncTree2 based on KO analysis in each cirrhotic cohort.

## Discussion

Advances in next-generation sequencing and metagenomic analyses have facilitated the investigation of the relationship between the human gut microbiome with individuals’ health and diseases. Although existing knowledge on the role of the gut microbiome in human health mainly comes from the analysis of diversity and the composition of the bacterial community, little is explored about those of phages [45]. To the best of our knowledge, this study was the first to explore the gut phageome of patients with liver diseases from a structural and functional perspective. Accordingly, sophisticated bioinformatic methods were adopted to characterize the phageome in fecal samples from cirrhotic patients with different etiologies in previously published studies. Moreover, we examined the total gut phageome of liver disorders at three levels, namely taxonomic classification, gene catalog construction, and KO analysis.

We analyzed gut phageome composition in cirrhotic patients with different etiologies using the custom method and detected no change in α- and β-diversities. This finding is in agreement with the findings in our previous study, indicating no evidence of a significant difference in bacterial community diversity. On the other hand, our comparative evaluation revealed some differences in the enteric phageome in different cohorts at the family level. This was significantly observed in Chinese ALC and NAFLD cohorts, in which the *Retroviridae* family was eliminated. Although this change was observed in the Chinese ALC cohort, no significant expansion of the taxonomic richness of *Retroviridae* phages was noticed in the Russian ALC samples. These findings are particularly interesting as the samples were collected from cirrhotic patients with a specific etiology (i.e., alcohol-induced cirrhosis) at two independent and geographically distinct regions. These findings would suggest geography, ethnicity, or lifestyle-based alterations in gut phageome communities. In a study by Pérez-Brocal et al. [46], phage strains from the family *Retroviridae* were more prevalent in IBD patients than in normal controls.

Interestingly, we found a phage signature in each etiology by determining individualized phage strains in each cohort. The comparison of the cohorts indicated that gut phageome could be unique at the strain level among cirrhotic patients concerning the etiology. For example, the results revealed that Pf1 phage belonging to the family *Inoviridae* was significantly overrepresented in NAFLD patients and that the other cohorts exhibited the total disappearance of this phage. Pf1 infects *Pseudomonas aeruginosa* strains and contributes to bacterial short-term evolution and virulence [47]. In another example, we indicated that two phage species in the genus *Lambdavirus* (namely *Escherichia* virus Lambda and *Escherichia* phage HK629) were more prevalent in the Chines HBV than the Chines ALC cohorts. As described, the human gut microbiome is expected to be strongly affected by the activity of the phage composition [17]. Accordingly, the increased prevalence of certain phages could be accompanied by specific dynamic interactions with their bacterial and human hosts [48–50]. Therefore, considering its specific etiology, such phage overrepresentation detected in our analysis may impose some alterations in the gut bacterial community in cirrhosis. More importantly, these data could be applied to detect whether the gut phageome signature is predictive of the diagnosis of liver cirrhosis induced by a specific etiology. However, some data in the present study suggested geography, ethnicity, or lifestyle-based alterations in the gut phageome of the cohorts. For example, compared to the other cohorts, the Chines cirrhotic cohorts phageome, regardless of their etiology, was characterized with remarkably overrepresented certain Enterobacteriaceae-specific phage groups, including those specific to *Klebsiella* and *Salmonella* spp. Another significant finding of our analysis was the significant differences in the richness of the gut phageome between the Russian ALC and the Chines ALC patients. Although the etiology of these patients was alcoholic cirrhosis, the Russian ALC exhibited a specific expansion of Lactobacillus and Leuconostoc-specific phages. In contrast, the predominant phages in the Chines ALC cohort were Enterobacteriaceae-specific strains.

Given that the taxonomic resolution of the gut phageome in liver cirrhosis with different etiologies can be similar and that their functionality can differ in several etiologies, we aimed to investigate the gut phageome in liver cirrhosis using a novel and comprehensive gut gene catalog for the 139 cirrhotic patients from different etiologies. We found that the core phageome of cirrhotic patients consists of at least 21 phage species, suggesting that these phages could be used to characterize cirrhosis by the gut phageome composition. However, these data do not account for temporal dynamics among the gut phageome, intestinal bacterial community, and the host. Accordingly, the findings on these phage categories are conservative and may need to be retested with temporal data from more subjects. Among these 21 phages, the newly-identified uncultured crAssphage (NC_024711.1) was the predominant strain in cirrhotic patients. Moreover, this phage is also known to be a highly prevalent phage in the unknown sequences of human fecal metagenomes [51]. Bacterial species belonging to the phylum Bacteroidetes, commonly found in gut mucosal microbiota, were predicted to be the host of phage crAssphage [52].

To the best of our knowledge, a phage gene catalog of distinct etiologies of liver cirrhosis was developed in this study for the first time. Remarkably, due to the lack of phage reference genomes and the high complexity of whole-community metagenomic sequencing data, it is challenging to discriminate phage and bacterial sequences in metagenomic datasets. In this study, the three different strategies, including CRISPR-based analysis, scaffolds generating by FastViromeExplorer, and scaffold generating by homologous proteins were applied to overcome the complications of constructing the gut phage catalog from whole-community metagenomic sequencing data. The largest majority of the identified phage scaffolds by our analysis were assigned as unclassified phages. The second majority of the identified phage scaffold in the current catalog was taxonomically related to the *Siphoviridae* family.

According to the results, we suggest that the significant discriminating features should be increased by using the functional analysis. As illustrated in Fig. 8, the PCA analysis results at both gene and KO levels revealed that KO might be a more accurate feature to predict the etiology of liver disorders; hence, more precise treatment and disease management protocols need to be developed and prescribed. KO can be considered as a more suitable discriminating feature in classifying different etiologies of liver cirrhosis, compared to taxonomic and even genomic features. Considerably, “K00986” and “K06905” were common in all six different groups. In this regard, the former is related to RNA-directed DNA polymerase, and the latter is related to an uncharacterized protein. Remarkably, each cohort has a significant number of KOs, for example, the NAFLD group has 36 dedicated KOs. Accordingly, the KO analysis could help more deeply investigate the exact function of the gut phageome, particularly in liver cirrhosis with different etiologies.

Our deep understanding of the composition and function of the gut phaegome in patients with liver diseases compared to healthy individuals can help guide us to find a unique “fingerprint marking” that may play a role in inter-individual phenotypic variation in disease development, prognosis, progression, and even response to treatment. In addition, further investigation could open new insights into the potential therapeutic benefits of phages in liver diseases. In the recent decade, some clinical trials have investigated the safety and efficacy of phage therapy for gastrointestinal diseases. Phage-based therapy for the treatment of patients with alcoholic liver disease was suggested previously by Duan et al. [53]. However, further clinical trials with a larger cohort are to validate these novel therapeutic approaches for patients with alcoholic cirrhosis.

## Conclusions

For decades, scientists have mainly disregarded the role of the gut microbiome as a potential modulator in the development and progression of gastrointestinal tract diseases, including liver diseases. In the current study, we found some specific taxonomic and functional alternations in the gut phageome among cirrhotic patients with distinct etiologies. *Pseudomonas* virus Pf1 phage and uncultured phage crAssphage have been eliminated in the NAFLD patients individually. However, our findings indicated that KEGG orthology analysis of the gut phageome can be a suitable discriminating feature compared to taxonomic and other genomic features in classifying different etiologies of liver cirrhosis In addition to focusing on the composition of phageome in liver diseases, investigating its functional features might also be insightful.

## Supplementary Information

**Additional file 1.** Scripts and Commands: All codes and scripts used in this study are available at https://github.com/Naseri1374/phageome-in-liver-cirrhosis. All information about each group including filtered genes (with FDR above 0.01), GhostKOALA and BlastKOALA results, specific KEGGs, pfam, cog, and eggnog results, and specific metabolic pathways are presented.

**Additional file 2.** Scripts and Commands: All codes and scripts used in this study are available at https://github.com/Naseri1374/phageome-in-liver-cirrhosis. All information about each group including filtered genes (with FDR above 0.01), GhostKOALA and BlastKOALA results, specific KEGGs, pfam, cog, and eggnog results, and specific metabolic pathways are presented.

**Additional file 3.** Scripts and Commands: All codes and scripts used in this study are available at https://github.com/Naseri1374/phageome-in-liver-cirrhosis. All information about each group including filtered genes (with FDR above 0.01), GhostKOALA and BlastKOALA results, specific KEGGs, pfam, cog, and eggnog results, and specific metabolic pathways are presented.

**Additional file 4.** Scripts and Commands: All codes and scripts used in this study are available at https://github.com/Naseri1374/phageome-in-liver-cirrhosis. All information about each group including filtered genes (with FDR above 0.01), GhostKOALA and BlastKOALA results, specific KEGGs, pfam, cog, and eggnog results, and specific metabolic pathways are presented

**Additional file 5.** Scripts and Commands: All codes and scripts used in this study are available at https://github.com/Naseri1374/phageome-in-liver-cirrhosis. All information about each group including filtered genes (with FDR above 0.01), GhostKOALA and BlastKOALA results, specific KEGGs, pfam, cog, and eggnog results, and specific metabolic pathways are presented

**Additional file 6.** Venn diagram results of KO analysis are shown.

**Additional file 7: Figure S1.** Functional annotation based on KO analysis performed by FuncTree2 in cirrhotic patients with various etiologies. The KEGG biologic categories are as follows: (1) cellular response to stress (crs); (2) Amino acid utilization biosynthesis metabolism (aau): methionine degradation (md), alanine, aspartate and glutamate metabolism (aag); (3) nucleic acid metabolism (nam): pyrimidine metabolism (pm); (4) carbohydrate Active enzyme(cae): glycoside hydrolase(gh); (5) fatty acid oxidation (fao): fatty acid alpha-oxidation (faao); (6) homoacetogenesis (ho): reductive acetyl coenzyme A pathway(racoa); (7) saccharide and derivated synthesis (sds): polysaccharide biosynthesis(pb), (8) transporters(tp): ABC transporter(abct); The KO frequency was determined and plotted (size = relative frequency of KOs in each plot).

## Data Availability

All metagenomics datasets used in this study were obtained from the NCBI short read archive (SRA) database (PRJEB6337; PRJNA373901; and PRJEB18041) generated in three previously-published microbiome studies [10, 24, 25].
