# Supplementary material for: Investigation and characterization of human gut phageome in advanced liver cirrhosis of defined etiologies
Source: Gut Pathog. 2022 Feb 15;14:9. doi: 10.1186/s13099-022-00482-4 (PMC8845349; doi:10.1186/s13099-022-00482-4)
Supplement: Supplementary file 7 — Additional file 7: Figure S1. Functional annotation based on KO analysis performed by FuncTree2 in cirrhotic patients with various etiologies. The KEGG biologic categories are as follows: (1) cellular response to stress (crs); (2) Amino acid utilization biosynthesis metabolism (aau): methionine degradation (md), alanine, aspartate and glutamate metabolism (aag); (3) nucleic acid metabolism (nam): pyrimidine metabolism (pm); (4) carbohydrate Active enzyme(cae): glycoside hydrolase(gh); (5) fatty acid oxidation (fao): fatty acid alpha-oxidation (faao); (6) homoacetogenesis (ho): reductive acetyl coenzyme A pathway(racoa); (7) saccharide and derivated synthesis (sds): polysaccharide biosynthesis(pb), (8) transporters(tp): ABC transporter(abct); The KO frequency was determined and plotted (size = relative frequency of KOs in each plot). [file 13099_2022_482_MOESM7_ESM.docx]

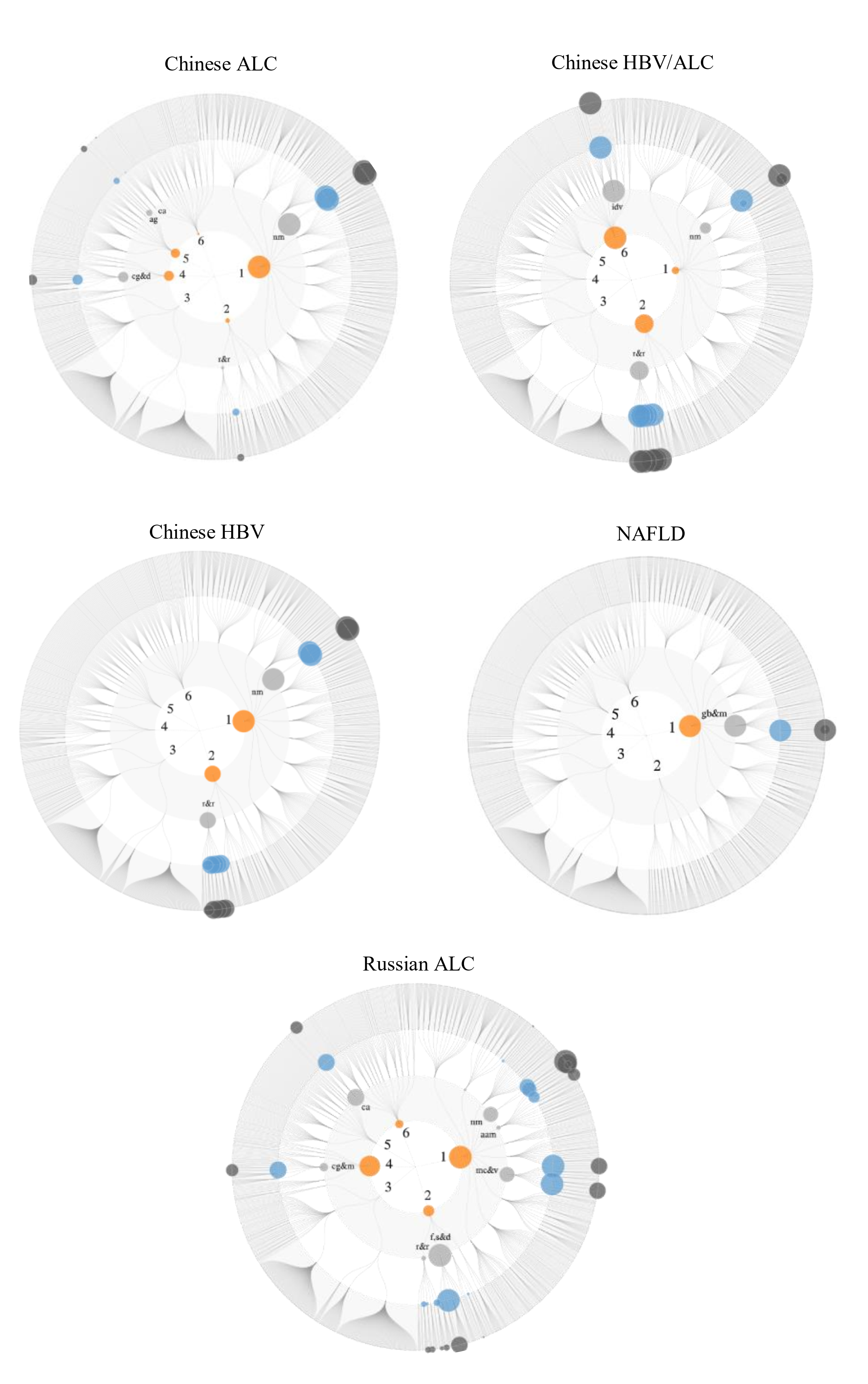


Figure S1: Functional annotation based on KO analysis performed by FuncTree2 in
cirrhotic patients with various etiologies. The KEGG biologic categories are as
follows: (1) cellular response to stress (crs); (2) Amino acid utilization biosynthesis
metabolism (aau): methionine degradation (md), alanine, aspartate and
glutamate metabolism (aag); (3) nucleic acid metabolism (nam): pyrimidine
metabolism (pm); (4) carbohydrate Active enzyme(cae): glycoside hydrolase(gh);
(5) fatty acid oxidation (fao): fatty acid alpha-oxidation (faao); (6)
homoacetogenesis (ho): reductive acetyl coenzyme A pathway(racoa); (7)
saccharide and derivated synthesis (sds): polysaccharide biosynthesis(pb), (8)
transporters(tp): ABC transporter(abct); The KO frequency was determined and
plotted (size = relative frequency of KOs in each plot).
